# Ventricular-subventricular zone stem cell niche adaptations in a mouse model of post-infectious hydrocephalus

**DOI:** 10.3389/fnins.2024.1429829

**Published:** 2024-07-31

**Authors:** Julianna Herman, Nicole Rittenhouse, Francesca Mandino, Mushirah Majid, Yuxiang Wang, Amelia Mezger, Aidan Kump, Sumeet Kadian, Evelyn M. R. Lake, Paulo H. Verardi, Joanne C. Conover

**Affiliations:** ^1^Department of Physiology and Neurobiology, University of Connecticut, Storrs, CT, United States; ^2^Department of Radiology and Biomedical Imaging, Yale University, New Haven, CT, United States; ^3^Department of Pathobiology and Veterinary Science, University of Connecticut, Storrs, CT, United States; ^4^Department of Biomedical Engineering, Yale University, New Haven, CT, United States; ^5^Wu Tsai Institute, Yale University, New Haven, CT, United States

**Keywords:** hydrocephalus, ventricular-subventricular zone, stem cells, ependymogenesis, influenza virus, post-infectious hydrocephalus, neurogenesis

## Abstract

Congenital post-infectious hydrocephalus (PIH) is a condition characterized by enlargement of the ventricular system, consequently imposing a burden on the associated stem cell niche, the ventricular-subventricular zone (V-SVZ). To investigate how the V-SVZ adapts in PIH, we developed a mouse model of influenza virus-induced PIH based on direct intracerebroventricular injection of mouse-adapted influenza virus at two distinct time points: embryonic day 16 (E16), when stem cells line the ventricle, and postnatal day 4 (P4), when an ependymal monolayer covers the ventricle surface and stem cells retain only a thin ventricle-contacting process. Global hydrocephalus with associated regions of astrogliosis along the lateral ventricle was found in 82% of the mice infected at P4. Increased ependymogenesis was observed at gliotic borders and throughout areas exhibiting intact ependyma based on tracking of newly divided cells. Additionally, in areas of intact ependyma, stem cell numbers were reduced; however, we found no significant reduction in new neurons reaching the olfactory bulb following onset of ventriculomegaly. At P4, injection of only the non-infectious viral component neuraminidase resulted in limited, region-specific ventriculomegaly due to absence of cell-to-cell transmission. In contrast, at E16 intracerebroventricular injection of influenza virus resulted in death at birth due to hypoxia and multiorgan hemorrhage, suggesting an age-dependent advantage in neonates, while the viral component neuraminidase resulted in minimal, or no, ventriculomegaly. In summary, we tracked acute adaptations of the V-SVZ stem cell niche following onset of ventriculomegaly and describe developmental changes that help mitigate the severity of congenital PIH.

## 1 Introduction

Within the developing brain, the lateral ventricle V-SVZ is responsible for providing neurons and glial cells (astrocytes and oligodendrocytes) to populate the developing structures of the forebrain (Noctor et al., 2008; Kriegstein and Alvarez-Buylla, 2009). From late embryonic to postnatal development V-SVZ stem cells become more restricted in their neurogenic capacity, primarily supplying newborn neurons for transit along the rostral migratory stream (RMS) to the olfactory bulb (Wichterle et al., 1999; Pencea et al., 2001; Kriegstein and Alvarez-Buylla, 2009; Conover and Shook, 2011; Sanai et al., 2011; Fujiwara and Cave, 2016; Paredes et al., 2016). Similarities between mouse and human V-SVZ neurogenesis exist during embryonic and early postnatal stages; however, humans have an additional neurogenic pathway to the ventromedial prefrontal cortex and a significant reduction of neurogenesis by 2-years, while mice have continued neurogenesis into old age (Sanai et al., 2011; Wang et al., 2011; Obernier and Alvarez-Buylla, 2019). In addition to neurogenesis, V-SVZ stem cells also generate a monolayer of ependymal cells (ependymogenesis), which line the ventricle surface and provide structural support to the ventricular system, fluid transport between the parenchyma and cerebrospinal fluid (CSF), and an immunological barrier (Del Bigio, 2010; Mishra and Teale, 2012; Roales-Bujan et al., 2012; Jiménez et al., 2014). Prior work identified mouse-human similarities in ependymal cell development and the organization of stem cells and ependymal cells along the lateral wall of the lateral ventricles (Spassky et al., 2005; Coletti et al., 2018). In brief, ependymogenesis begins caudally from embryonic day 13 (E13) in the mouse, which equates to human ependymogenesis at gestational week 18 (GW18), and proceeds rostrally so that by postnatal day 7 (P7), the lateral wall of the lateral ventricle is mainly covered with a mature ependyma, which equates to human ependymal coverage found at GW34 (McAllister et al., 2017; Coletti et al., 2018). Similar cytoarchitectural organization, including ‘pinwheel’ units, newly generated ependymal cells surround clusters of stem cell processes (cell bodies of stem cells are relegated to the SVZ), is found along the lateral wall of both humans and mice (Spassky et al., 2005; Mirzadeh et al., 2008). These overt developmental similarities support the use of mouse models for investigations of congenital diseases that impact the V-SVZ, such as post-infectious hydrocephalus.

Post-infectious hydrocephalus (enlargement of the ventricles) can arise due to CNS infection. Specifically, influenza virus-associated encephalitis has been observed with hydrocephalus development in both children and adults (Meijer et al., 2016; Albaker et al., 2019) and maternal influenza has been associated with congenital hydrocephalus (Krous et al., 1978; Luteijn et al., 2014; Mak et al., 2018), implicating influenza virus as a risk factor not only for ventricle enlargement and associated sequelae, but also for disruption of ventricular-subventricular zone (V-SVZ) stem cell niche functions and concomitant developmental disorders. The age of onset strongly dictates the severity of neurological impairment and overall prognosis; however, the pathological sequence that results in post-infectious hydrocephalus (PIH) and the effect on the associated V-SVZ stem cell niche remain unclear. One major aspect of disease progression is that ependymal denudation at the ventricle surface is replaced by glial scarring and ventriculomegaly (discussed in McAllister, 2012). During influenza viral infection, the superficial glycoprotein hemagglutinin binds to α2,6- and 2,3-linked sialic acid receptors typically of epithelial cells (Matrosovich et al., 2013; Benam et al., 2019). A second viral component, neuraminidase, cleaves sialic acids from the surface of infected cells to release viral progeny from the cell surface (Air and Laver, 1989; McAuley et al., 2019). Ependymal cells contain sialic acids moieties recognized by neuraminidase (Murlidharan et al., 2015) and our lab (Luo et al., 2008; Shook et al., 2014) and others (Grondona et al., 1996; Granados-Durán et al., 2016; Fernández-Arjona, 2021) have found that neuraminidase injected into the lateral ventricles can cleave linkages between cells leading to denudation of the ependyma and ventriculomegaly.

To examine age-dependent response to influenza virus infection, we developed a mouse model of PIH based on intracerebroventricular injection of mouse-adapted influenza virus. This allowed us to examine the direct impact of influenza virus infection on the lateral ventricle stem cell niche and its functions in repair and neurogenesis. Since early embryonic and neonatal organization of the lateral ventricle lining in mouse closely approximates human fetal and neonatal development, we restricted our studies to two developmental timepoints that display distinct cytoarchitectural organization of stem cells at the lateral ventricle surface—embryonic day 16 (E16), when stem cells line the ventricle surface before the generation of the ependymal cell barrier, and postnatal day 4 (P4), after the ependyma monolayer is laid down and the remaining stem cells only contact the ventricle surface with an apical process. These timepoints allowed us to identify and separate vulnerabilities along the ventricle wall based on cell composition and organizational maturity at the time of infection. Additionally, we correlated the extent of ventricle enlargement with V-SVZ stem cell niche disruptions and adaptations to provide a more complete analysis of the data. We included examination of neuraminidase, a non-infectious component of the influenza virus, that others (Grondona et al., 1996; Granados-Durán et al., 2016; Fernández-Arjona, 2021) and our laboratory (Luo et al., 2008; Shook et al., 2014) found leads to ventriculomegaly through disruption of ependymal cells and resultant ventricle surface astrogliosis. Together, our studies reveal that the time of influenza virus, or neuraminidase, introduction is critical, in part, due to the changing cellular composition of the lateral ventricle lining, with V-SVZ compensation occurring in a P4 mouse model of PIH.

## 2 Materials and methods

### 2.1 Animals

Male and female CD-1 mice (*Mus musculus*) (Charles River Laboratories, Wilmington, MA, United States) were used in all studies. Mice were housed on a 12-h light/dark cycle with *ad libitum* food and water. Cages were individually ventilated. Embryonic mice were purchased from Charles River Laboratories, and we followed colony maintenance strategies based on *Breeding Strategies for Maintaining Colonies of Laboratory Mice*. *A Jackson Laboratory Resource Manual*. Housing, handling, care, and processing of the animals were carried out in accordance with regulations approved by the Institutional Animal Care and Use Committee of the University of Connecticut and Yale University (MRI data collection) and followed the National Institute of Health Guide for the Care and Use of Laboratory Animals.

### 2.2 Influenza virus and heat inactivation

Mouse-adapted influenza A virus, A/WSN/1933 (H1N1), was sourced from BEI Resources, NIAID, NIH (catalog number NR-3688, titered in MDCK cells). The number of infectious units used (280 TCID50) was determined by pilot experiments, where rates of survival and ventriculomegaly were optimized following intracerebroventricular injections in P4 mice. To heat-inactivate A/WSN/1933 influenza virus, frozen virus was thawed, mixed, and incubated in a 70°C thermal block for 30-min. Virus inactivation was confirmed according to Jonges et al. (2010) by culturing inactivated samples on MDCK cells and looking at lack of cytopathic effects, as well as by quantitative reverse transcription PCR (RT-qPCR) analysis for lack of influenza virus mRNA production in infected MDCK cells.

### 2.3 Neuraminidase

Neuraminidase from influenza A virus A/New Caledonia/20/1999 (H1N1, 250 ng/μL) was sourced from BEI Resources (catalog number NR-43779). The appropriate amount necessary to generate ventriculomegaly was determined by pilot experiments where expansion of the lateral ventricles was assessed (see Section 2.9) in P4 mice. The administered dose was selected when ventriculomegaly was replicated in roughly 50% of intracerebroventricularly-injected P4 mice.

### 2.4 Intracerebroventricular injections

#### 2.4.1 Embryonic *in utero* injections

Intracerebroventricular injections were performed on E16 mice using *in-utero* injection, as previously described (Walantus et al., 2007; LoTurco et al., 2009). Timed-pregnant females were anesthetized with ketamine (100–120 mg/kg, i.p.) and xylazine (7–8 mg/kg, i.p.) according to body weight, followed by subcutaneous administration of buprenorphine (0.5 mg/mL). Following an incision of the abdominal cavity, uterine horns were exposed, and the right lateral ventricle of the exposed embryos was injected with influenza virus (0.5 μL) at a dose of 140 TCID_50_, or with neuraminidase (0.5 μL, 125 ng). Sterilized 0.9% saline (0.5 μL) or heat-inactivated influenza virus (0.5 μL) were injected as controls. A microinjector (Picospritzer III; General Valve, Fairfield, NJ) outfitted with a pulled glass capillary needle, prepared with a Narishige PC-10 micropipette puller, delivered the injection dose. The uterine horns were then replaced, and the incision was sutured closed. Mice were monitored for recovery following surgery while on a heating pad and returned to cage once fully awake and capable of movement.

#### 2.4.2 Postnatal injections

P4 mice were anesthetized by placement on ice until a toe pinch response could not be elicited. For P4 injections, influenza virus (1 μL, 280 TCID_50_), neuraminidase (1 μL, 250 ng), sterilized 0.9% saline (1 μL) or heat-inactivated influenza virus (1 μL, see above) were injected directly into the right lateral ventricle using a 26-gauge Hamilton brand needle (CAL87900). Based on the Allen Brain Atlas, the following coordinates, x: 0.8 mm; y: 1.5 mm; z: 1.5 mm, were used. Mouse pups were placed on a heating pad directly after injection until movement returned, and then rubbed with nesting material before being returned to their dam.

### 2.5 5-Ethynyl-2-deoxyuridine (EdU)

To label newly dividing cells, EdU (150 mg/kg, from a 10 mg/mL working solution) was injected i.p. 3-days after the initial intracerebroventricular injection. MRI data collection was performed at P26-29 following intracerebroventricular injection. For tissue collection, mice were perfused with 0.9% saline, brains were removed and postfixed in 4% paraformaldehyde (PFA) overnight followed by three 5-min washes in phosphate buffered saline (1X PBS). Brains were sectioned coronally (50 μm), and EdU was visualized using the Click-It EdU Alexa Fluor-647 Imaging Kit (Thermo Fisher Scientific, C10340) according to the manufacturer’s instructions.

### 2.6 Lateral ventricle whole mount preparations

Mice were anesthetized with isoflurane, then transcardially perfused with 0.9% saline. The extracted brains were dissected into whole mounts of the lateral ventricles, as previously described (Mirzadeh et al., 2008). Whole mount preparations were fixed overnight in 4% PFA at 4°C. All wholemounts were washed 3 times for 10-min in PBS (1X) before immunostaining. Blocking solution was then applied [10% Normal Donkey Serum (NDS, Jackson Immuno), 1% Triton X-100 (1% TX) in PBS (1X)] for 1-h and tissue wholemounts were incubated with the following primary antibodies for 48-h with 1% TX and 10% NDS in PBS (1X) at 4°C: goat anti-influenza A virus, (RRID: AB_775660, 1:300); rabbit anti-γ-tubulin, (Sigma-Aldrich Cat# SAB4503045, RRID:AB_10747615, 1:800); mouse anti-β-catenin, (BD Biosciences Cat# 610154, RRID:AB_397555, 1:250); rat anti-GFAP (Thermo Fisher Scientific Cat# 13–0300, RRID:AB_2532994) and wholemounts were washed 4 times for 5-min with PBS (1X), then incubated in secondary antibody solution: donkey anti-rabbit 546 (Thermo Fisher Scientific Cat# A10040, RRID:AB_2534016), RRID:AB_2535792, 1:500, donkey anti-mouse 647 (Thermo Fisher Scientific Cat# A-31571, RRID:AB_162542), donkey anti-goat 405 (Thermo Fisher Scientific Cat# A48259, RRID:AB_2890272), donkey anti-rat 488 (Thermo Fisher Scientific Cat# A48269, RRID:AB_2893137) in 1% TX and 10% NDS in PBS (1X) at 4°C for 48-h. Tissue wholemounts were then washed with PBS (1X), and those samples that included EdU were treated with a Click-It EdU Kit for EdU visualization (Invitrogen, C10340). Whole mounts were mounted on slides using Aqua-PolyMount (Polysciences Inc.), dried for a minimum of 48-h, then imaged using Leica Sp8 confocal microscope. All antibodies used in this study were previously validated by our group and others; the expression patterns were as previously seen and referenced (Mirzadeh et al., 2010; Coletti et al., 2018).

### 2.7 V-SVZ analysis: astrogliosis, stem cell quantification, and ependymogenesis

Following MRI data collection, wholemount preparations were made of the lateral wall of the lateral ventricles (see Section 2.6) and the tissue was imaged on a Leica Sp8 confocal microscope. To observe the effects of influenza virus and neuraminidase at the ventricle surface, we identified regions of astrogliosis by GFAP immunostaining and ependymal cell denudation, where intact ependymal cells were defined by multiple γ-tubulin^+^ puncta and regular β-catenin expression. For single cell analysis, images were collected from regions without ependymal denudation. The ventricle was divided into a grid of 18 sections (approximately 1 × 10^5^ mm^2^ per square), with 9 squares in the rostral section and 9 squares in the caudal section. From each of these 18 regions, images were taken as a z-stack (2.1 × 10^4^ mm^2^). Stem cells were identified by z-stacks, with a single γ-tubulin^+^ puncta at the cell’s apical surface and GFAP^+^ staining throughout the cell body. Pinwheel regenerative units were identified as a ring of ependymal cells surrounding a cluster of stem cells. Ependymal cells, identified by multiple γ-tubulin^+^ puncta with surrounding β-catenin, were observed for EdU^+^ nuclei, using z-stack tracing.

### 2.8 Analysis of neurogenesis

Following EdU injection (see above) and MRI data collection (see below), olfactory bulbs were collected, stored in 4% PFA for a minimum of 2-days and the right olfactory bulbs were sectioned (50 μm) with a vibratome. Sections were blocked in 10% NDS in PBS (1X) with 0.1% TX for 1-h. Sections were then incubated at 4°C with guinea pig anti-doublecortin (DCX) (Millipore Cat# AB2253, RRID:AB_1586992, 1:1000) primary antibodies. Sections were then washed four times with PBS (1X) and incubated overnight at 4°C with donkey secondary antibodies: Fluor 405 (Sigma-Aldrich Cat# SAB4600230, RRID:AB_2637046, 1:500) in 10% NDS in PBS (1X) with 0.1% TX. Following a PBS (1X) wash, EdU was detected using a Click-IT EdU Cell Proliferation Kit (Invitrogen, C10340), following the manufacturer’s instructions. Sections were mounted onto slides using Aqua-PolyMount (Polysciences Inc.) and left for at least 24-h prior to imaging. Slides were coded (for blinded study) and every other section was imaged using a Leica SP8 confocal microscope for a mosaic z-stack. ImageJ was used to minimize background noise and allow clear visualization of each individual nucleus. Final images were then analyzed using ZEISS Arivis Vision4D software with 2D Viewer. Within this software, the analysis panel used included “Blob Finder” and “Export Object Features.” The settings of the analysis panel were custom ROI set to all bounds and all planes. The settings under Blob Finder were consistently set to a length of 5 μm; 20% probability threshold; and 55% split probability. The resulting output data points that were 1–3 μm were considered as noise and excluded from quantification and the remaining data points were evaluated to ensure EdU authenticity.

### 2.9 Lateral ventricle volume assessment: coronal section reconstructions

Lateral ventricle volumes were assessed by coronal section reconstructions when early time point analysis was required (24- and 72-h) post-intracerebroventricular injections. Mouse brains that had been injected with saline or influenza virus were perfused with 0.9% saline and 4% PFA, then set to fixed in an 8:2 solution of 4% PFA:PBS (1X) for a minimum of 24-h at 4°C. Vibratome sections (50 μm) were collected beginning at the rostral-most opening of the ventricles (1.42 mm from bregma, Allen Brain Atlas) and ending at the third ventricle opening (−1.34 mm from bregma, Allen Brain Atlas). Every other brain section was blocked with 500 μL blocking solution (10% NDS in PBS with 0.1% TX) per slide for a minimum of 30-min. Following removal of blocking solution, sections were incubated at 4°C overnight. Coronal sections were stained with mouse anti-s100β (Proteintech Cat# 66616-1-Ig, RRID:AB_2881976, 1:250) and goat anti-GFAP (Thermo Fisher Scientific Cat# PA5-143587, RRID:AB_2942816, 1:250). Slides were rinsed 3 times with PBS (1X), leaving the last wash on the slides for a minimum of 2-h at 4°C. This was followed by 3 more PBS washes. Sections were then incubated overnight at 4°C with the appropriate donkey secondary antibody Alexa Fluor 546 (Thermo Fisher Scientific Cat# A10036, RRID:AB_2534012, 1:500), Alexa Fluor 647 (Thermo Fisher Scientific Cat# A-21447, RRID:AB_2535864, 1:500) and Alexa Fluor 488 (Thermo Fisher Scientific Cat# A-21202, RRID:AB_141607, 1:500) in PBS (1X) and 10% NDS and then rinsed 3 times with PBS (1X). DAPI solution (Thermo Fisher Scientific Cat# D1306, RRID:AB_2629482) was applied for a minimum of 30-min and after a final 3 rinses with PBS (1X), the slides were covered using Aqua-PolyMount (Polysciences Inc.) and coverslipped. Sectional brain volumes and lateral ventricle volumes were traced using an Apotome Imager.M2 microscope (ZEISS) and Neurolucida software (MBF Biosciences), then reconstructed using Neurolucida Explorer (MBF Biosciences) for calculations of ventricle volumes.

### 2.10 Lateral ventricle volume assessment: MRI and data acquisition

An 11.7 preclinical (Bruker, Billerica, MA) scanner was used to collect structural MRI data using an in-house built saddle coil for whole brain coverage. Mice were free-breathing but anesthetized with isoflurane (4% induction, ~1–1.5% maintenance, in a medical air/O_2_ 50–50% mixture). The MRI data were acquired using ParaVision version 6.0.1 software. Body temperature was maintained with a circulating water bath at 36.6–37°C.

#### 2.10.1 Data exclusion

For our study, mice were excluded based on established criteria: observable hemorrhaging during injection protocol or use in initial pilot studies to determine appropriate dosages (excluded n = 42 of total n = 150). Of the mice included in this work, 44 were female and 64 were male.

#### 2.10.2 Structural MRI

Two types of structural images are acquired for data registration purposes and volumetric analyses. (1) A whole-brain isotropic 3D image using a multi-spin-multi-echo (MSME) sequence, with 0.2 × 0.2 × 0.2 mm^3^ resolution, TR/TE of 7,000/20 ms, 78 slices, and 2 averages, field-of-view 17.6 × 8.8 mm^2^ image size 88×44 for a total scan length of 10-min and 16-s. (2) A Rapid Imaging with Refocused Echoes (RARE) scan, whole-brain coverage, RARE factor = 30, TR/TE of 3,000/70 ms and resolution of 0.120 × 0.120 × 0.120 mm^3^ isotropic, field-of-view 13.2 × 9.6 × 14.4 mm^3^, image size 110 × 80 × 120, for a total scan length of 13-min and 6-s.

#### 2.10.3 Data processing and 3D renderings

Data from both structural scans were analyzed using customized modules within the in-house built software Bioimage Suite (BIS) Web, which is freely available online (Yale MRRC). Specifically, the RARE structural images were thresholded based on signal intensity within the ventricles compared to surrounding brain tissue, to allow for manual segmentation of the regions of interest. MR images of the cerebral ventricles were filled and traced using the BIS Image editor. Volume was quantified as pixels, then converted to mm^3^. 3D images of the generated object maps were created using the “Viewer Control” feature, then exported.

## 3 Experimental design and statistical analysis

All statistical analysis were performed in RStudio. The minimum level of significance for all tests was *p* < 0.05. 24-h volumetric data are reported as mean ± s.e.m. To identify sex differences in right ventricle volume in our P4 and E16 groups, we used unpaired *t*-tests to compare lateral ventricle volumes of male and female mice in each group. MRI data collection compared saline, heat-inactivated influenza virus, neuraminidase, and influenza virus injected right ventricle volumes and consistently demonstrated no significant difference between the sexes in any case (*p* = 0.12; *p* = 0.81; *p* = 0.70; *p* = 0.55, respectively).

To assess statistical significance in stem cell counts between influenza virus and saline cases, Welch’s two sample *t-*test was performed comparing the difference in percent of stem cells per pinwheel between saline and influenza virus groups (1–2 stem cells per unit, *p* = 0.00016, 3–5 stem cells per unit; *p* = 0.00067; 5–10 stem cells per unit, *p* = 0.0014). To compare the total number of pinwheel units between saline and influenza virus groups, a Welch’s two sample *t-*test was performed (*p* = 0.28). A Welch’s two sample *t-*test was also applied to compare total stem cell numbers between saline and influenza virus groups (*p* = 0.0026).

To compare means in the neurogenesis studies, a one-way ANOVA was performed (*p* = 0.86). We ran a Welch’s two sample *t*-test to compare the means of saline to heat-inactivated influenza virus (*p* = 0.63), the means of saline to influenza virus (*p* = 0.78), and the means of heat-inactivated influenza virus to influenza virus (*p* = 0.75).

## 4 Results

### 4.1 H1N1 infects stem cells and ependymal cells along the ventricle surface

To understand the impact of hydrocephalus on the V-SVZ stem cell niche during embryonic and neonatal development, we sought to develop a consistent, high efficacy mouse model of PIH by introducing influenza virus directly into the lateral ventricle. Two timepoints were selected based on the changing developmental status of the ventricle lining: embryonic day 16 (E16), a stage when the lateral wall of the lateral ventricle consists predominantly of stem cells with only a few immature ependymal cells, and postnatal day 4 (P4), a stage when the ventricular surface is populated primarily by mature ependymal cells surrounding the apical processes of the remaining stem cells in a pinwheel conformation, with stem cell bodies relegated to the SVZ (Spassky et al., 2005; Mirzadeh et al., 2008; Coletti et al., 2018).

**Figure 1:**
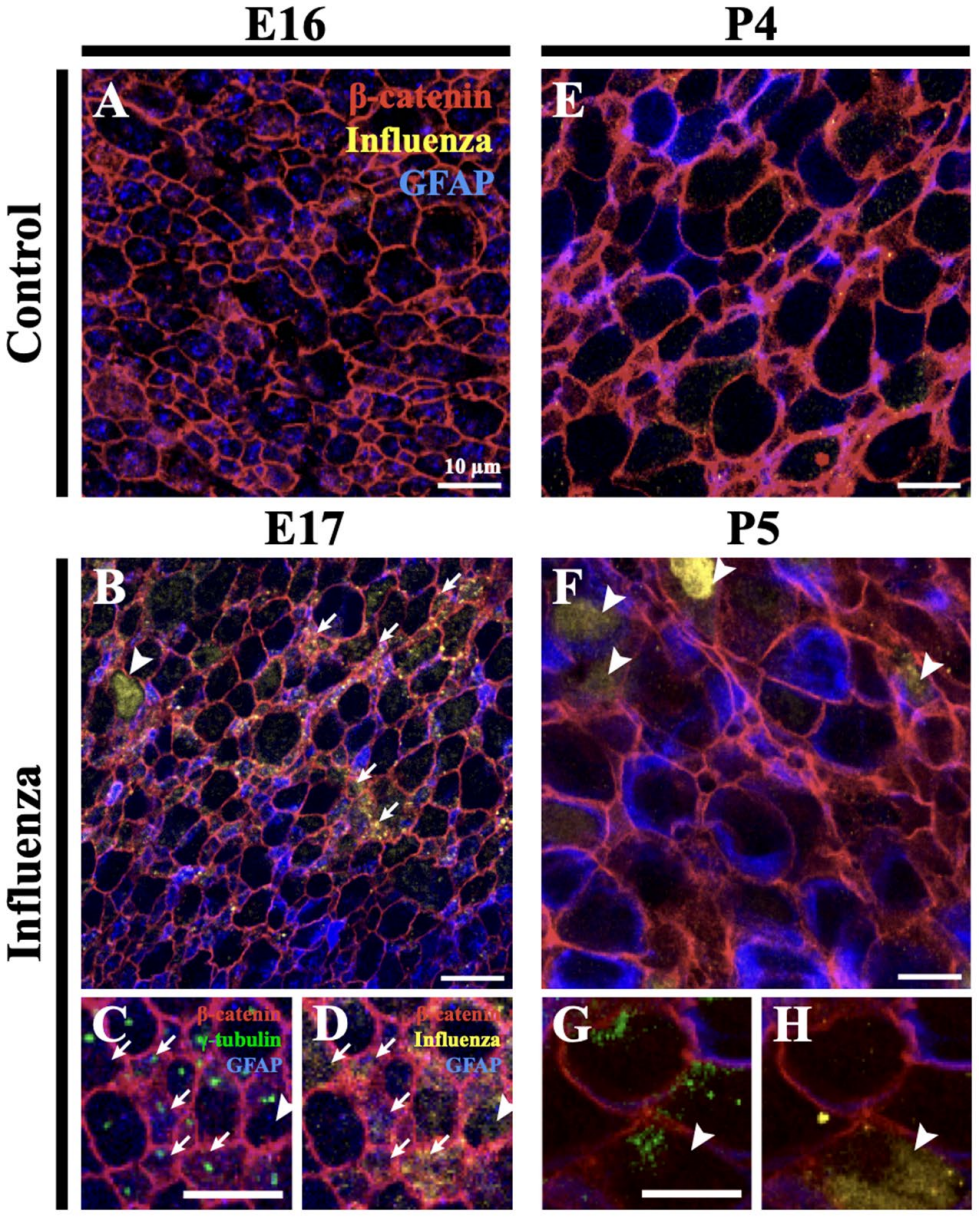
Influenza virus infects stem cells and ependymal cells of the V-SVZ. **(A)** *En face* view of a control mouse (E16) lateral wall of the lateral ventricle whole mount stained for influenza virus. **(B)** Following influenza virus injection at E16, influenza virus was observed 24-h post-injection (E17) in stem cells (arrows) and immature ependymal cells (arrowhead, n = 8). **(C,D)** Increased magnification and channel separation shows stem cells, identified by a β-catenin membrane, GFAP and a single γ-tubulin^+^ basal body (single puncta, white arrow), and immature ependymal cells, identified by a β-catenin membrane and 2–5 γ-tubulin^+^ basal bodies (two or more puncta, white arrowhead), were targets of influenza virus infection. **(E,F)** At P4, influenza virus was not detected in control lateral ventricle whole mounts **(E)** but was detected 24-h post-influenza virus infection in stem cells (not shown) and mature ependymal cells **(F)** (white arrowheads, *n*=3). **(G,H)** Increased magnification and channel separation shows influenza virus within a mature ependymal cell.

To assess infectivity along the lateral ventricle surface, we performed unilateral, right intracerebroventricular injections of mouse-adapted H1N1 influenza A virus (280 TCID_50,_ New Caledonia, A/WSN/33) at E16 and P4. At 24-h post-injection, mice were perfused with 0.9% saline, brains were collected, and whole mounts of the right lateral ventricle were prepared, then fixed in 4% paraformaldehyde (PFA) (Doetsch et al., 1999; Mirzadeh et al., 2008; Coletti et al., 2018). Stem cells were identified based on GFAP^+^ immunostaining and the presence of a single apical cilium identified by a γ-tubulin^+^ basal body at the ventricle surface. Ependymal cells were delineated by β-catenin^+^ cell membranes and the presence of multiple basal bodies of cilia (γ-tubulin^+^ clusters) at their apical surface. For the E16 injection timepoint, we detected influenza virus 24-h post-infection in both stem cells and the few immature ependymal cells found along the ventricle surface; no influenza virus was detected in age-matched (uninfected) controls (Figures 1A–D). For the P4 injection timepoint, we detected influenza virus 24-h post-injection of influenza virus in both mature ependymal cells and stem cells, but not in age-matched controls (Figures 1E–H). This established that influenza virus infected both stem cells and ependymal cells along the ventricle surface following intracerebroventricular injections of H1N1 influenza virus.

### 4.2 Ventriculomegaly develops in influenza virus-injected neonatal mice

**Figure 2:**
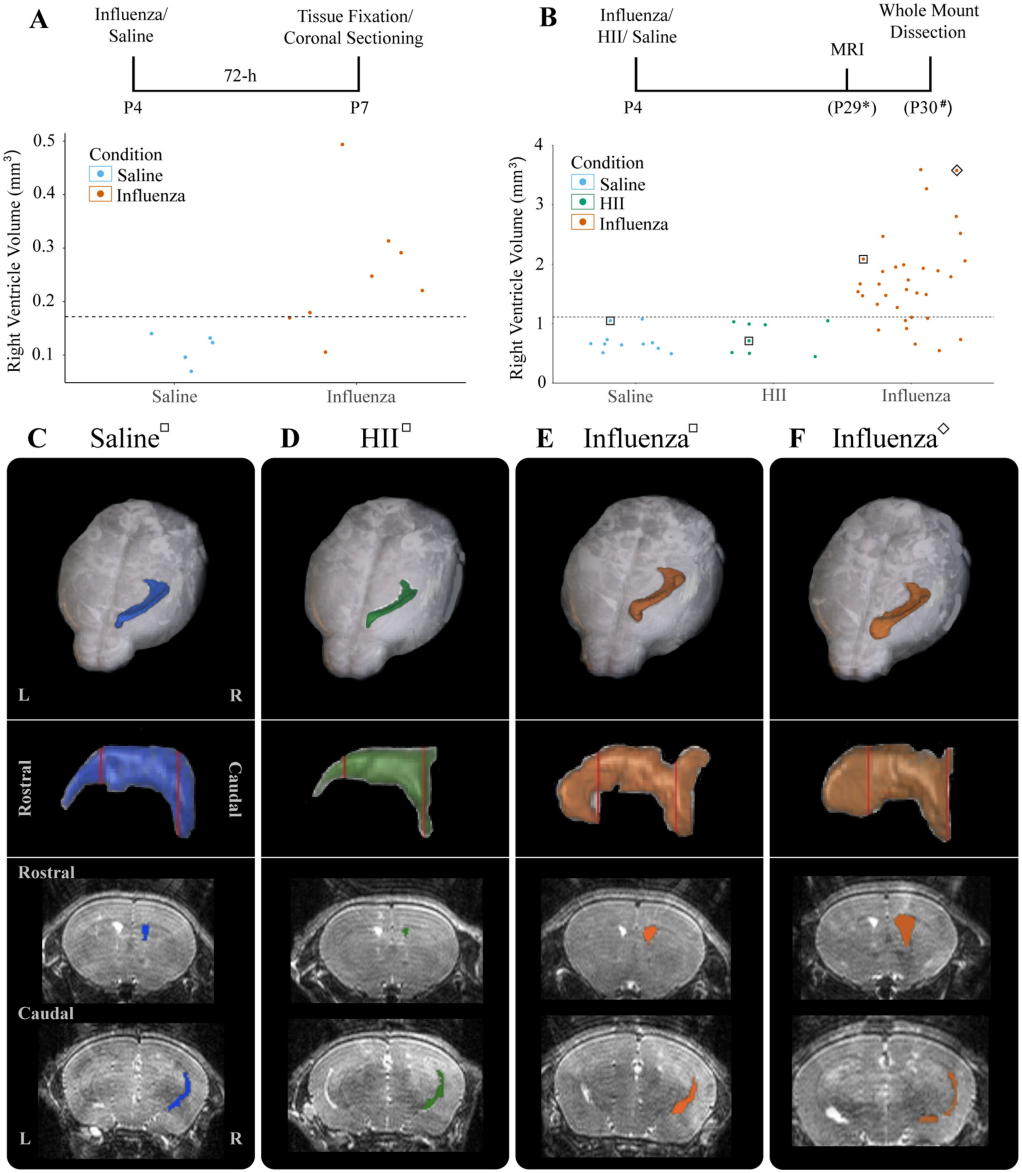
Ventricular enlargement was observed in P4 influenza virus-injected mice. **(A)** Timeline and graph for 72-h assessment of ventricle volumes for saline- and influenza virus-injected right lateral ventricles. The threshold for ventricle enlargement (0.17 mm^3^) is indicated by a dashed horizontal line. **(B)** Timeline and graph for 30-day assessment of ventricle volumes for saline-, HII-, and influenza virus-injected mice (* indicates range of P25-32, # indicates a range of P28-31). The threshold for ventriculomegaly (1.11 mm^3^) is indicated by a dashed horizontal line. **(C–F)** Top panels show a dorsal view of a 3D right lateral ventricle MRI reconstruction for saline **(C)**, HII **(D)**, and influenza virus **(E,F)** injected mice with their respective ventricle volumes indicated in **(B)**, denoted by squares around dots indicating individual mice. Middle panels show a sagittal view of each reconstructed right lateral ventricle. Red vertical lines indicate the locations of the coronal sections below.

At P4, we established that an intracerebroventricular injection (1 μL) of mouse-adapted influenza virus (A/WSN/1933, H1N1) at a dose of 280 TCID_50_ consistently resulted in ventriculomegaly, with full patency, by P30 with no significant difference between the sexes (*p* = 0.55) (see Section 3). To establish a timeline for onset of lateral ventricular expansion and, we performed unilateral (right ventricle) intracerebroventricular injections of influenza virus H1N1 or saline (control) in P4 neonatal mouse pups and examined ventricle volumes 24-h and 72-h post-injection. Brains were perfused with saline, then with 4% PFA, and fixed overnight in an 8:2 solution of 4% PFA:PBS before forebrains were sectioned coronally. Tissue sections were immunostained to identify ependymal cells (s100β^+^, γ-tubulin^+^ basal body clusters) lining the ventricle surface. ZEISS Apotome Vision 2.0 Neurolucida software was used to perform ventricle tracing and volume assessments. Ventricles with a volume greater than +2SD of the control mean were defined as having ventriculomegaly. At a 24-h timepoint after intraventricular injection of either influenza virus or saline (control), no ventricle enlargement was detected (saline, 0.15 ± 0.018 mm^3^; influenza virus, 0.14 ± 0.004 mm^3^). However, 72-h post-injection, 82% of influenza virus-injected mice showed ventricular enlargement greater than +2SD of the mean of saline controls (calculated as 0.17 mm^3^, Figure 2A). This indicated that following intracerebroventricular influenza virus introduction at P4 onset of ventriculomegaly occurred between 24–72 h.

To determine effects of influenza virus injections within the postnatal period, P4 mice were unilaterally (right lateral ventricle) injected with influenza virus. Saline and heat-inactivated influenza virus (HII) (see Section 2) injections were used as vehicle and inactive viral particle-specific controls, respectively. Ventricle volumes were assessed at P26-29 after each intracerebroventricular injection using an 11.7 T preclinical magnet. Following MRI data collection, mice were perfused within 72-h and wholemounts of the lateral wall for the lateral ventricle were prepared (Figure 2B, timeline). Using BioImage Suite software (Yale MRRC), ventricle volumes were calculated, and 3D images based on the volumetric map of each mouse ventricle were generated (Figures 2B–F). The threshold for hydrocephalus was determined as +2SD above the mean of the control, which was calculated as a ventricle volume greater than 1.11 mm^3^ (Figure 2B, dotted line). Within the influenza virus group, 82% showed enlarged ventricles; no saline or HII cases fell above the threshold for ventriculomegaly (Figure 2B). Together these studies established a working model for post-infectious hydrocephalus (PIH).

### 4.3 Regions of ventricular expansion are associated with astrogliosis

**Figure 3:**
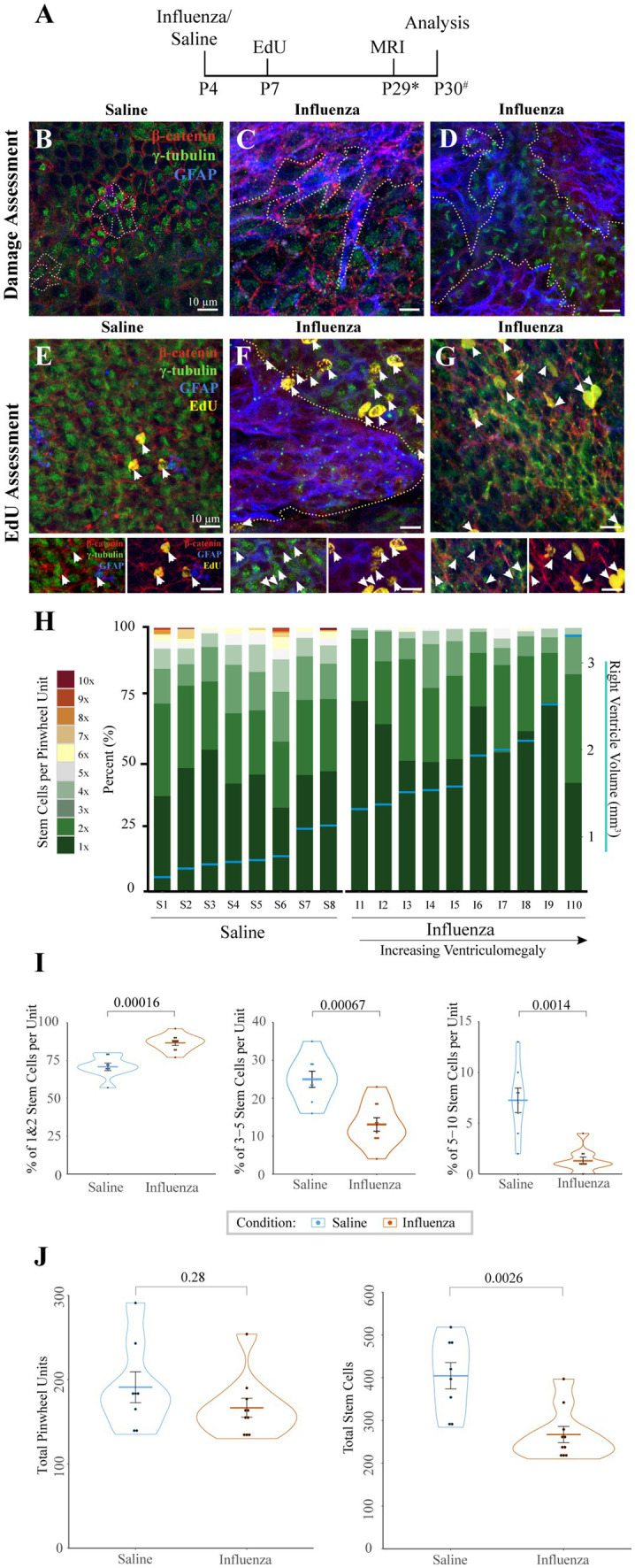
Cellular response to influenza virus following P4 infection. **(A)** Timeline for P4 injections of influenza virus or saline (* indicates range of P25-32, # indicates a range of P28-31). **(B)** Whole mount preparations of the ventricle surface from saline-injected mice shows an intact ependymal layer with pinwheels (ependymal cells surrounding individual stem cell apical processes, dotted outline). **(C,D)** Whereas the ventricle surface following influenza virus injection reveals large areas of astrogliosis (dotted outlines) that disrupt areas of intact ependyma in both rostral **(C)** and caudal **(D)** regions of the ventricle. **(E)** Following an EdU injection at P7, the ventricle surface of saline injected mice shows few EdU^+^ ependymal cells (white arrows, channel separation is shown below). **(F)** However, after injection of influenza virus, the ventricle surface has increased numbers of EdU^+^ ependymal cells (white arrows) associated with astrogliotic borders (outlined with dotted line, channel separation shown below). **(G)** Increased numbers of EdU^+^ ependymal cells (white arrows, channel separation shown below) were also found in non-gliotic regions along the ventricle surface. **(H)** Stem cell numbers were counted in regions without astrogliosis for each mouse; the percentage of total pinwheels with 1–10 stem cells per pinwheel unit is indicated. Eight saline-injected and ten influenza virus-injected mice, with ventriculomegaly, were assessed and ordered by ventricle volume (x-axis), designated by horizontal blue lines with corresponding volumes shown on the right y-axis. **(I)** Statistical analysis of **(H)** indicates that influenza virus-injected ventricles have a significantly higher percentage of pinwheels with only 1–2 stem cells (*p* = 0.00016) and a corresponding significantly lower percentage of pinwheels with 3–5 (*p* = 0.00067) and 5–10 (*p* = 0.0014) stem cells (unpaired t-test). **(J)** Statistical analysis of **(H)** indicates that influenza virus-injected ventricles do not have significantly different numbers of total pinwheel units across the surface of the ventricle (left; *p* = 0.28), but total stem cell numbers are significantly depleted in influenza virus-injected mice (right; *p* = 0.0026).

We assessed the entire right (injected) lateral ventricle surface for changes in ependymal and stem cell organization (Figure 3). Following intracerebroventricular injection at P4 and MRI data collection to allow us to determine ventriculomegaly status (Figure 3A), we examined the ventricle surface of control (Figure 3B) and experimental mice with ventriculomegaly (Figures 3C,D). Wholemounts of the right lateral ventricle wall from control mice showed an intact ependymal cell lining with stem cells arranged in pinwheel units (Figure 3B, dotted lines). However, following influenza virus injection, the ventricular surface revealed large areas of astrogliosis (GFAP^+^ astrocytes demarcated by dotted lines, Figures 3C,D) that neighbored areas of intact ependyma.

### 4.4 Astrogliotic regions are associated with increased new ependymogenesis

To assess cell division patterns within the V-SVZ following intracerebroventricular injections, mice were intraperitoneally injected with EdU 72-h post-saline or influenza virus injection (Figure 3A). Whole mount preparations (Doetsch et al., 1999; Mirzadeh et al., 2008; Coletti et al., 2018) of the right lateral ventricle (injected) wall were made approximately one day following MRI data collection. Whole mounts were immunostained to identify stem cells (GFAP^+^, γ-tubulin^+^ single apical cilia), ependymal cells (γ-tubulin^+^ multiple apical cilia, β-catenin^+^ border), astrocytes (GFAP^+^), and newly dividing cells (EdU^+^). We collected images of the entire surface of the lateral wall of the ventricle using Sp8 confocal microscopy. Normal low levels of ependymogenesis were seen along the lateral wall of our saline-injected group (Figure 3E). However, in influenza virus-injected mice, regions of ventricle expansion where there was ependymal denudation replaced by astrogliosis showed increased levels of EdU^+^ ependymal cells associated with the astrogliotic boundaries (Figure 3F). Additionally, increased levels for EdU^+^ ependymal cells were also found throughout the expanded ventricle wall in regions with an intact ependyma (Figure 3G).

### 4.5 Stem cells per pinwheel unit are depleted in influenza virus induced PIH

Stem cells along the lateral wall of the lateral ventricle are organized into pinwheel units, with several stem cell processes per pinwheel. To determine if stem cell numbers differed in intact regions of the lateral ventricle between saline and influenza virus-injected mice, we quantified both the total number of stem cells and stem cells per pinwheel unit, based on an 18-region grid system distributed across the surface of the rostral and caudal lateral wall of the lateral ventricle. From each of these regions, images of intact ependyma were taken as a z-stack (see Sections 2, 2.7). In mice with ventriculomegaly, gliotic areas were devoid of stem cells and therefore were not assessed. Eight saline-injected mice and ten influenza virus-injected hydrocephalic mice were examined using this method. The number of stem cells per pinwheel unit were counted based on z-stack reconstructions and presented graphically as the percentage of pinwheels that had differing numbers of stem cells (Figure 3H, left y-axis). Associated ventricle volumes (indicated by blue horizontal lines, Figure 3H, right y-axis) were also included. We found that the influenza virus group had a greater number of pinwheels with only 1–2 stem cells (average of 87% of all pinwheel units) compared to saline-injected ventricles (average of 71%, *p* = 0.00012) (Figure 3I). Conversely, we found that the influenza virus group had significantly fewer pinwheels with 3–5 stem cells (13% influenza virus versus saline control average of 25%, *p* = 0.00067) and 5–10 stem cells (1% influenza virus versus 7% saline control, *p* = 0.0014). Interestingly, we observed no significant difference in the total number of pinwheel units between saline and influenza virus injected mice in the 18 regions examined (*p* = 0.28) (Figure 3J, left), but instead we observed a significant reduction in the global total stem cell number in our influenza virus group (*p* = 0.0026) (Figure 3J, right). These data indicate that while influenza virus injected mice show an overall reduction in stem cell number, the large numbers of stem cells per pinwheel unit typically found during the postnatal period allows retention of pinwheel units but with fewer stem cells.

### 4.6 Olfactory bulb neurogenesis was not affected in our neonatal PIH model

**Figure 4:**
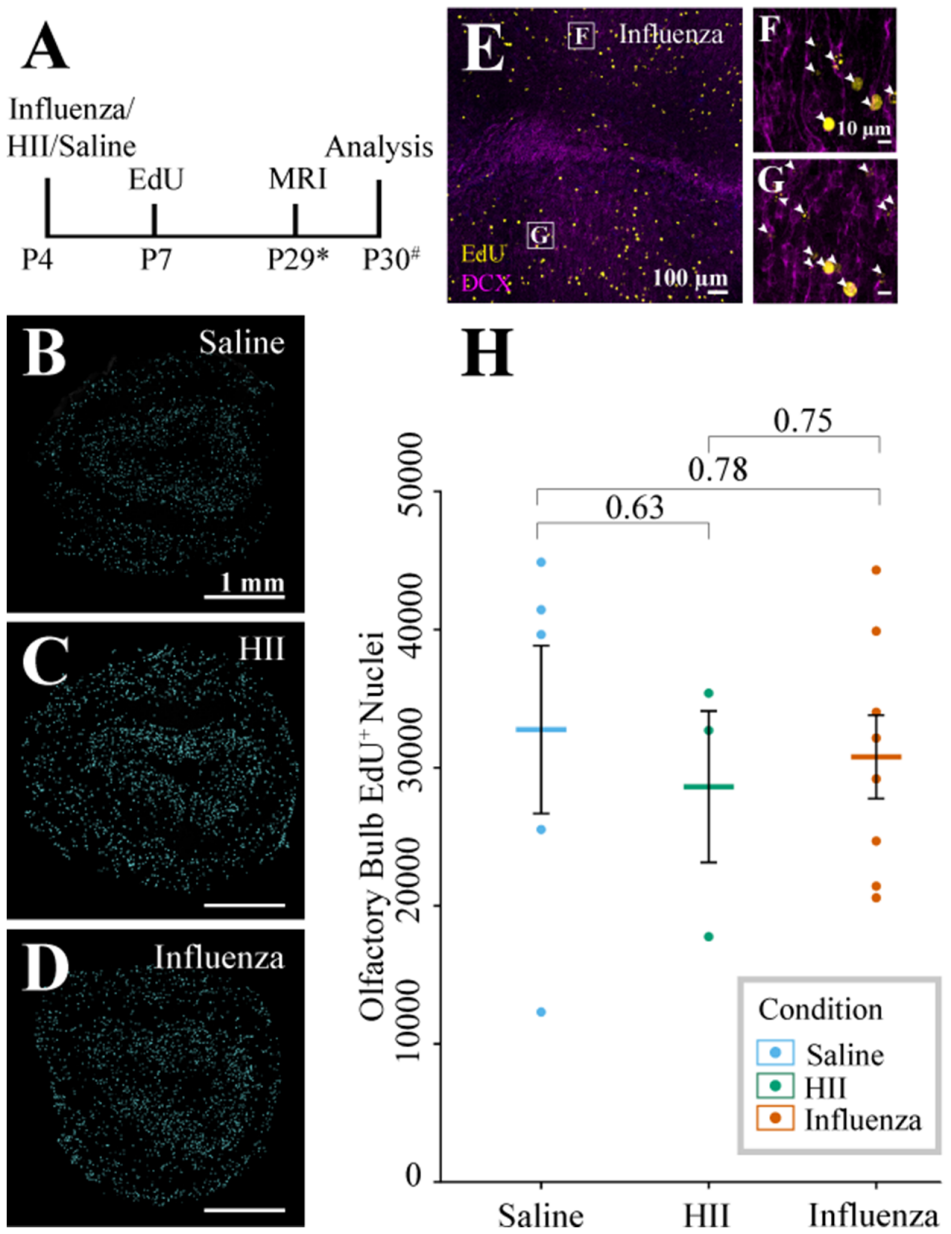
Olfactory bulb neurogenesis assessment. **(A)** Timeline of the procedure (* indicates range of P25-32, # indicates a range of P28-31). **(B–D)** Representative sections post-processing used for quantification of EdU^+^ cells following saline, HII, and influenza virus injections. **(E–G)** Representative olfactory bulb images used for quantification show DCX and EdU co-labeling (arrowheads, boxes in **E** indicate magnified images in **F** and **G**). **(H)** Olfactory bulb EdU^+^ counts indicate no significant differences between groups.

During neonatal development and throughout the life of the mouse, V-SVZ stem cells continuously generate new neurons that migrate from the V-SVZ through the forebrain via the RMS to their destination in the olfactory bulb (see reviews: Lledo et al., 2008; Sun et al., 2010). Based on the early onset of ventriculomegaly (Figure 2A) and decreases in stem cell number observed at the ventricle surface following influenza virus exposure (Figures 3H–J), we hypothesized that a reduction in stem cell numbers may affect downstream neurogenesis. To explore whether ventriculomegaly onset affected the number of neuroblasts that reach the olfactory bulb, mice were injected with EdU 72-h following the initial intracerebroventricular injection of influenza virus, heat-inactivated influenza virus (HII) or saline (Figure 4A). Newly generated neuroblasts take 5–9 days to traverse the RMS and enter the olfactory bulb (Luskin, 1993; Lois et al., 1996; Sun et al., 2010; James et al., 2011; Martinez-Molina et al., 2011; Conover and Todd, 2017; Chen et al., 2023). Therefore, we allowed time for migration to be complete and MRI data collection. Olfactory bulbs were collected within 48-h post-MR imaging with fixation in 4% PFA overnight. The right olfactory bulbs (injected side) were coronally sectioned (50 μm), stained using antibodies for EdU (progeny of dividing cells) and DCX (migratory neuroblasts) and imaged using a Leica Sp8 confocal microscope. Images were randomized following image acquisition and were then counted in a blinded analysis. Quantification of EdU^+^ nuclei (Figures 4B–D) was performed on stained olfactory bulb sections (Figures 4E–G) using Arivis Vision 4D software (ZEISS). We found no significant difference in EdU^+^ cell counts between the control groups (saline and HII) and the influenza virus-injected mice that developed ventriculomegaly [ANOVA *p* = 0.86; saline, influenza virus *t*-test *p* = 0.78; HII, influenza virus *t*-test *p* = 0.75] (Figure 4H). These data disproved our hypothesis and indicated that despite development of ventriculomegaly, neuroblasts were able to populate the olfactory bulb at equivalent levels to what was found in control mice (saline-and HII-injected mice).

### 4.7 Neonatal neuraminidase injection results in limited, region-specific ventricle expansion

**Figure 5:**
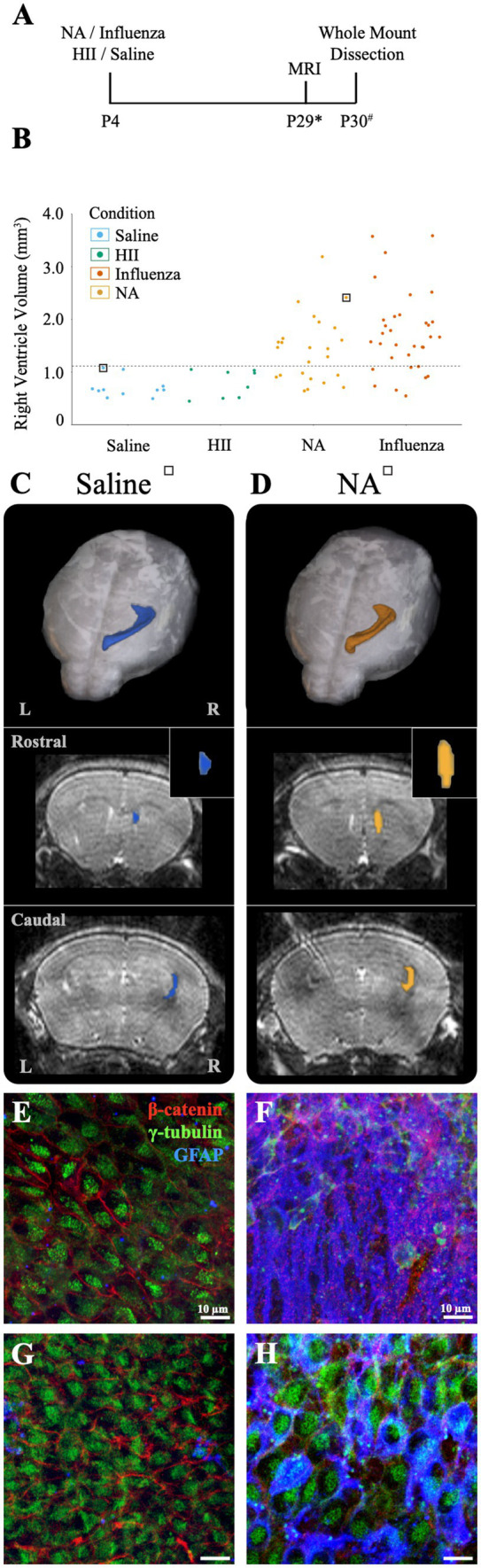
Intracerebroventricular injection of neuraminidase (NA) at P4 resulted in limited, localized ventricle expansion. **(A)** Procedural timeline for NA, influenza virus, HII, and saline injections and associated analysis (* indicates range of P25-32, # indicates a range of P28-31). **(B)** Ventricle volumes following NA injection are now combined with saline, HII and influenza virus volumes presented earlier with the dashed line indicating the threshold for ventriculomegaly (1.11 mm^3^). Saline and HII injections did not result in ventricle enlargement, whereas NA and influenza virus injections resulted in 71 and 82% ventricle enlargement, respectively. **(C,D)** Top panels show dorsal 3D views of the injected, right lateral ventricle following MRI reconstruction. The middle and bottom panels show rostral and caudal views, respectively, with the insert highlighting the rostral increase in volume in the NA brain. Mice used in **(C,D)** are indicated with a square around a dot in **(B)**. **(E,F)** Representative images from the rostral ventricle wall show intact lateral ventricle walls in the saline injected ventricle but astrogliosis associated with rostral expansion in the NA injected ventricle, respectively. **(G,H)** Representative caudal images from the saline injected ventricle and the NA injected ventricle reveal an intact ependyma, but an upregulation of GFAP in ependymal cells was observed **(H)**.

Neuraminidase, a glycoprotein found on the surface of influenza virus, is implicated in cleavage of sialic acid at the apical pole of epithelial cell types (Air and Laver, 1989; Shtyrya et al., 2009; McAuley et al., 2019). In the brain, neuraminidase has been shown to cause ependymal cell denudation and ventriculomegaly when administered in the lateral ventricular system (Luo et al., 2006; Shook et al., 2014; Fernández-Arjona, 2021). For the purposes of our study, neuraminidase provides a univariant model for influenza virus-induced hydrocephalus since it is just one component of the virus but is incapable of cell-to-cell transmission. Neonatal P4 mice were injected with 1 μL neuraminidase (250 ng/μL, A/New Caledonia/20/1999) and MRI data collection was performed approximately 30-days later (range: P25-32). Whole mounts of the injected, right ventricle wall were prepared within 72-h of MRI data collection (Figure 5A). Ventricle volumes were determined using BioImage Suite software and whole mount preparations of the ventricle wall were immunostained and imaged, as described above. Of the neuraminidase-injected ventricles, 71% showed region-specific expansion that qualified for ventriculomegaly (Figure 5B), frequently with only the rostral portion of the lateral ventricle presenting evident expansion when compared to the saline-injected control brains (Figure 5C versus Figure 5D). Global expansion of the lateral ventricle, as seen with influenza virus injections, was not observed. Ventricle wall whole mounts were immunostained to identify stem cells (GFAP^+^, γ-tubulin^+^ single apical cilium), ependymal cells (γ-tubulin^+^ multiple apical cilia, β-catenin^+^ cell membrane), and astrocytes (GFAP^+^). Regions of intact ependymal cells were detected throughout the wall in saline-injected mice (Figures 5E,G). In neuraminidase-injected mice, areas of regional expansion showed astrogliosis as indicated by increased GFAP expression and ependymal cell loss (Figure 5F). However, regions that did not correlate with ventricle expansion, as determined by MRI, revealed an intact ependyma; however, we detected frequent upregulation of GFAP within intact ependymal cells (Figure 5H).

### 4.8 Perinatal death follows embryonic injections of influenza virus, while neuraminidase injections result in low level or no ventriculomegaly

**Figure 6:**
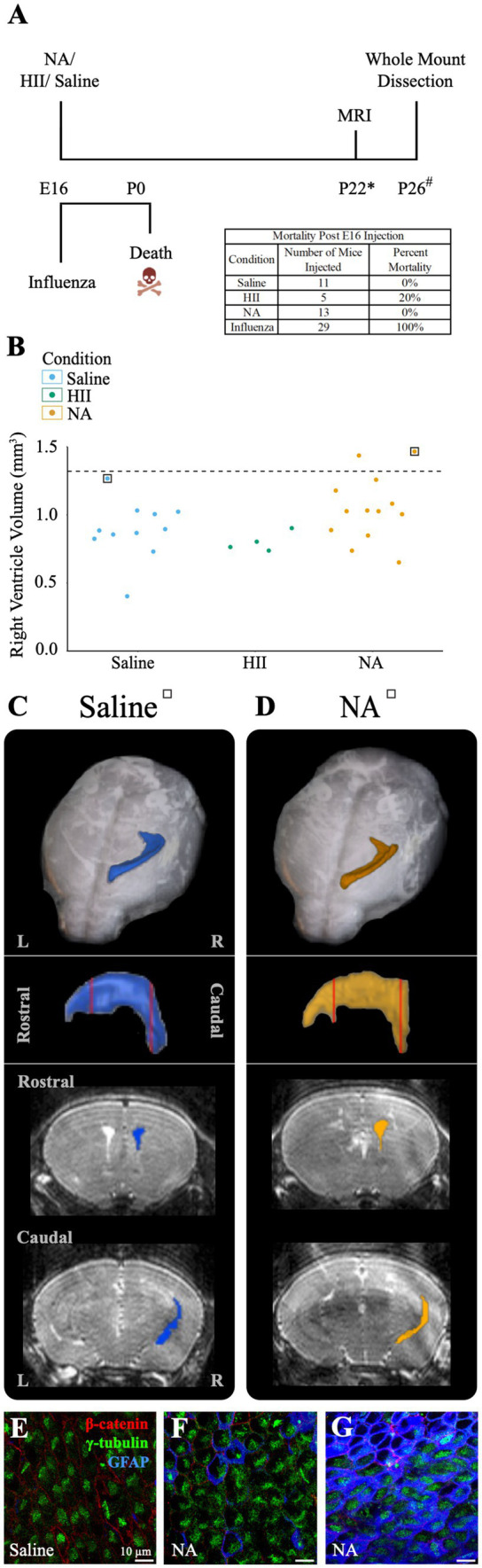
E16 injections of influenza virus result in death, while E16 NA injections result in little or no ventricle enlargement. **(A)** Procedural timeline for injections in E16 embryos is shown. The table indicates mortality percentage for all experimental conditions. Death is observed at birth following *in utero* influenza virus injections. (* indicates range of 23 ± 4, # indicates a range of P20-23). **(B)** Right ventricle volumes for saline, HII, and NA injected E16 embryos. The dashed line indicates the threshold for ventriculomegaly (1.34 mm^3^). **(C,D)** Top panels show dorsal views of the right lateral ventricle MRI reconstruction from saline and NA injected mice (for each mouse its associated ventricle volumes is indicated in **(B)** by a square). Middle panels show sagittal view with red vertical lines indicating the locations of the rostral and caudal coronal sections shown below (bottom panels). **(E,F)** The saline-injected and rostral NA-injected ventricle surfaces reveal an intact ependymal wall. **(G)** The caudal region of the NA-injected ventricle reveals an intact ependymal surface but shows an upregulation of GFAP in ependymal cells.

At E16, neural stem cells take up an average of 83% of the total lateral ventricle surface area, with the remainder occupied by newly formed, immature ependymal cells (Spassky et al., 2005; Coletti et al., 2018). We used E16 mice to assess the effect of influenza virus on an immature ventricle lining versus the newly established ependyma found in our P4 model (above). *In utero* intracerebroventricular injections (0.5 μL) of H1N1 influenza virus (140 TCID_50_) were performed. At this dose we observed 100% mortality at birth (n = 29). Similarly, when we made a 100-fold dilution (1.4 TCID_50_) of the viral titer we also found 100% mortality at birth (n = 8). In contrast, following intracerebroventricular injections of saline or neuraminidase, all mice were healthy with 0% mortality (The HII control group had 1 death occurring 4-days after birth) (Figure 6A). Post-mortem tissue analysis performed on deceased, influenza virus injected (E16) mouse pups revealed multiorgan hemorrhage, tissue necrosis and inflammation in multiple organs with cause of death determined to be vascular perturbations and hypoxia (pathological necroscopy, Dr. Neha Mishra, Connecticut Veterinary Medical Diagnostic Laboratory), likely due to disseminated influenza virus infection in the immunologically immature embryonic stage, and perhaps promoted by the fragility of local blood vessels (reviewed in: Saunders et al., 2012; Wälchli et al., 2023).

To determine whether ventriculomegaly developed when E16 embryos were intracerebroventricularly injected with neuraminidase, we assessed 28 E16 pups approximately 25-days post-injection (range P19-P27) using MRI and collected whole mount preparations of the injected ventricle linings. Ventricular volume assessment revealed that only two neuraminidase-injected embryos (7% of all injected mice) qualified as ventriculomegaly (1.44 mm^3^, 1.47 mm^3^); the threshold for hydrocephalus was determined to be 1.34 mm^3^ based on saline control data. (Figure 6B). MRI 3D reconstructions for saline-and hydrocephalic neuraminidase-injected mice right ventricles are shown (Figures 6C,D). In contrast to the fatal effect of influenza virus on E16 embryos, injection of neuraminidase caused only limited ventricle expansion and no visible ependymal denudation or astrogliosis (Figures 6E,F). However, we did observe an increased GFAP presence within ependymal cells in the slightly enlarged caudal-most region of one of the neuraminidase-injected ventricles (Figure 6G), similar to what we found in Figure 5H, and as reported by others in injury studies (Namiki, 1999; Ceruti et al., 2009; Rodriguez-Jimenez et al., 2023) and noted by our lab in the aging V-SVZ (Luo et al., 2008).

## 5 Discussion

### 5.1 Assessment of congenital post-infectious hydrocephalus (PIH)

Hydrocephalus is one of the oldest known neurological disorders, and yet is still the leading cause of pediatric brain surgery today (Aschoff et al., 1999; Tully and Dobyns, 2014). Model-based strategies to aid our understanding of hydrocephalus have largely prioritized assessment of CSF impairment (reviewed in Duy et al., 2023). Mechanical obstruction of the ventricular system or induction of genetic mutations as in *hyh* mice, *Htx* rats, and ciliary or stem cell defects (Jiménez et al., 2001; Mashayekhi et al., 2002; Guerra et al., 2015; Duy et al., 2019, 2022; García-Bonilla et al., 2020; Henzi et al., 2020; Xu et al., 2021) among other methods, have been used to examine obstructive or non-communicating hydrocephalus (discussed in McAllister, 2012; Munch et al., 2023), while many examinations of communicative hydrocephalus have relied on injection of a bacterially sourced viral component, neuraminidase, which is known to denude the ependymal cell monolayer, resulting in ventriculomegaly (Grondona et al., 1996; Shook et al., 2012; Granados-Durãn, 2015; Fernández-Arjona, 2021). Exposure to live virus has also been used in hydrocephalus modeling to profile protein expression and establish viral transmissibility during ventriculomegaly onset (Krous et al., 1978; Hayashi et al., 1986; Mockus et al., 2020; Robert et al., 2023). These models, while critical for our understanding of the different modes of hydrocephalus onset and progression, do not fully address alterations to and adaptations of the critical V-SVZ stem cell niche.

Influenza virus has been linked to human hydrocephalus (Krous et al., 1978; Luteijn et al., 2014; Khasawneh et al., 2018; Mak et al., 2018). Here, we focused on a congenital PIH mouse model specifically caused by mouse-specific H1N1 influenza virus. Based on previously profiled cell populations of the V-SVZ (Coletti et al., 2018), we selected timepoints associated with critical cytoarchitectural changes at the ventricle surface—stem cell dominance along the lateral ventricle surface for the embryonic timepoint (E16) and the formation of an ependymal cell barrier layer at the neonatal timepoint (P4). Previous work from our lab demonstrated that the lateral ventricle lining in embryonic mice closely approximates what is found during the second trimester in human infants – a time when hydrocephalus is frequently diagnosed. Similarly, the mouse neonatal timepoint approximated the third trimester in humans, with ependymal cells reaching complete maturation across the ventricular surface (Spassky et al., 2005; Coletti et al., 2018). Mouse-adapted H1N1 influenza A virus was selected as it has been previously implicated in neonatal hydrocephalus development and noted for its neurovirulent potential, but had not been assessed for its impact on local stem cell populations or their functions; critical aspects of neurotropic disease onset (Aronsson et al., 2002; Luteijn et al., 2014; Wang et al., 2014; Green et al., 2018; Mátrai et al., 2022). We also used viral neuraminidase as a non-infectious comparative model to our PIH model. Neuraminidase has historically been used in assessments of communicative hydrocephalus by others (Grondona et al., 1996; Granados-Durãn, 2015; Granados-Durán et al., 2016; Fernández-Arjona, 2019, 2021) and our lab (Luo et al., 2008; Shook et al., 2014), and thereby allows comparison between the multivariant effects of influenza virus versus a single, non-infectious component of influenza virus.

### 5.2 Developmental timepoint dictates distinct response

Based on the developmental timepoints used, we found perinatal lethality following intracerebroventricular injection of influenza virus at E16 in all cases (even at a 100-fold lower dose) versus long-term survival of mice with ventriculomegaly following infection at P4. This supported our hypothesis that a mature ependyma contributes to improved resilience, as well as the immunologically immature embryonic stage and, perhaps, the tenuousness of local blood vessels (reviewed in: Saunders et al., 2012; Wälchli et al., 2023). A review by Rasmussen and work by others (Lieberman et al., 2011; Rasmussen et al., 2012) discussed influenza exposure in pregnancy and concluded that in humans, increased ease of viral propagation in the second or third trimester leads to increased mortality in infants. In addition to hydrocephalus development, maternal influenza infection has shown heightened risk of neurodevelopmental disorders including schizophrenia, autism spectrum disorder, and attention deficit disorder (Patterson, 2002; Shi et al., 2005; Meyer et al., 2009; Fatemi et al., 2017; Egorova et al., 2024). While fetal brain damage has been theorized to play a major role in these outcomes; little is known about the virus’ impact on neurodevelopment, or its more direct effect on the cells at the V-SVZ (see San Martín-González et al., 2023). Our work demonstrates the susceptibility of embryonic tissue to influenza virus infection and provides additional context for the response of the V-SVZ to studies that have previously only considered the effect of virus on extrinsic cell signaling (Mockus et al., 2020; Robert et al., 2023) or prioritized establishing hydrocephalus as a result of viral infection within the brain (Krous et al., 1978; Aronsson et al., 2002).

**Figure 7:**
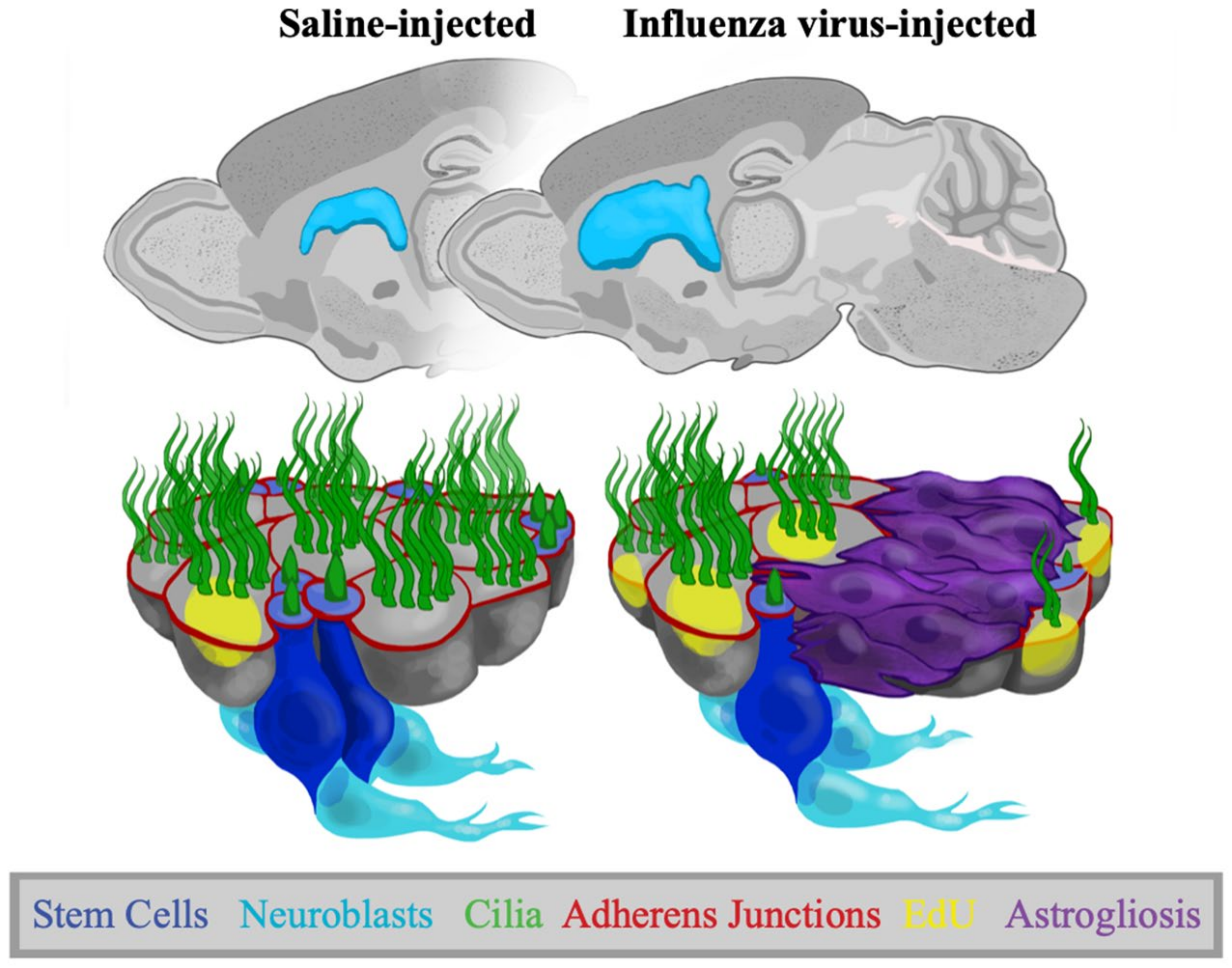
Adaptations to the V-SVZ in a postnatal PIH mouse model. Representative sagittal views of P30 control and PIH mouse brains. Schematics show the ventricle lining following saline and influenza virus injection, where there is increased astrogliosis, increased EdU^+^ ependymal cells, reduced stem cell numbers, but no change in neurogenesis following influenza virus infection.

Since mice developed ventriculomegaly (with full patency) following neonatal infection, we could assess changes to the ventricle surface and V-SVZ niche functions (summarized in Figure 7). In all mice showing ventriculomegaly, we found large areas of astrogliosis along the lateral ventricle surface in line with what others and our lab had reported in both human and mouse studies of ventricular enlargement (Domínguez-Pinos et al., 2005; Johanson et al., 2011; Shook et al., 2014; Conover and Todd, 2017; Varela, 2020). We evaluated stem cell numbers in the areas of intact ependyma (without gliosis), revealing a reduction when compared to the ventricle lining of control mice. We also noted an increase in the number of EdU^+^ ependymal cells, indicating ependymogenesis is upregulated at the onset of ventriculomegaly (between P5-P7). EdU^+^ ependymal cells were found both directly adjacent to astrogliotic borders and in regions with uninterrupted ependymal cells. Additionally, we found that neurogenesis, the number of new neurons migrating from the V-SVZ to the olfactory bulb, was not altered in our P4 PIH mouse model. Specifically, to evaluate neurogenesis following onset of ventriculomegaly, mice were administered EdU at P7 to label newly generated neuroblasts. We counted EdU^+^ neuroblasts 21–24 days following EdU injection, providing ample time for migration and distribution within the olfactory bulb, and observed no significant difference between saline-injected and influenza virus-injected groups. Neuroblast migration takes approximately 5–9 days to reach the olfactory bulb via the RMS (Luskin, 1993; Lois et al., 1996; Sun et al., 2010; James et al., 2011; Martinez-Molina et al., 2011; Todd et al., 2017).

The increase in ependymogenesis and the maintenance of neurogenesis during the period of ventricle enlargement suggests V-SVZ stem cells are actively dividing at the onset of ventriculomegaly. We found stem cell numbers per pinwheel unit and thereby stem cell numbers overall were decreased in intact regions of the lateral ventricle in mice with influenza virus-induced ventriculomegaly—pinwheel units were maintained but stem cell processes per pinwheel were reduced. These findings are reminiscent of our finding in the aging V-SVZ, where we observed decreased numbers of stem cells per pinwheel but an activation of remaining stem cells that maintained neurogenesis levels (Shook et al., 2012). Therefore, the destruction of stem cells in gliotic regions may lead to the activation of remaining stem cell populations to address ependymogenesis and neurogenesis. Notably, proliferation of neural stem cells is known to occur in response to traumatic brain injury (Benner et al., 2013; Ye et al., 2014; Wang et al., 2016), in line with our observation of neurogenesis and increased ependymogenesis. While these processes may be beneficial in the early stages of ventricle enlargement, they may result in a decrease of neurogenic potential later in life. A similar immediate compensatory effect followed by neurogenesis decline has been reported when assessing the effect of early life stress on neurogenesis in rats (Suri et al., 2013; Koutmani and Karalis, 2015). Alternatively, it is also possible that increased division of transit amplifying cells are responsible for the maintenance of neurogenesis in our model (Quiñones-Hinojosa et al., 2006; Kriegstein and Alvarez-Buylla, 2009; Li et al., 2012). Ultimately, long-term assessments of our PIH model coupled with stem cell lineage tracing will provide mechanistic insight into neurogenesis alterations in a compromised V-SVZ.

## 6 Conclusion

Following onset of ventriculomegaly, the immediate areal loss of ependymal cells is addressed by astrogliosis; however, regions of the ventricle surface with an intact ependyma remain and stem cells in these regions maintain neurogenesis and promote ependymogenesis. The generation and maintenance of an ependymal barrier appears critical not only for ventricular system functions but also the maintenance of a functional V-SVZ stem cell niche.

## Data availability statement

The original contributions presented in the study are included in the article/supplementary material, further inquiries can be directed to the corresponding author.

## Ethics statement

The animal study was approved by Institutional Animal Care and Use Committee. The study was conducted in accordance with the local legislation and institutional requirements.

## Author contributions

JH: Conceptualization, Data curation, Formal analysis, Funding acquisition, Investigation, Methodology, Project administration, Supervision, Validation, Visualization, Writing – original draft, Writing – review & editing. NR: Conceptualization, Formal analysis, Investigation, Validation, Visualization, Writing – review & editing, Writing – original draft. FM: Data curation, Investigation, Software, Visualization, Writing – original draft, Writing – review & editing. MM: Investigation, Methodology, Writing – review & editing. YW: Conceptualization, Investigation, Supervision, Validation, Writing – original draft, Writing – review & editing. AM: Investigation, Validation, Visualization, Writing – review & editing. AK: Investigation, Visualization, Writing – review & editing. SK: Investigation, Visualization, Writing – review & editing. EL: Conceptualization, Investigation, Methodology, Software, Supervision, Visualization, Writing – original draft, Writing – review & editing. PV: Conceptualization, Formal analysis, Investigation, Methodology, Project administration, Resources, Supervision, Validation, Writing – original draft, Writing – review & editing. JC: Conceptualization, Formal analysis, Funding acquisition, Investigation, Methodology, Project administration, Resources, Supervision, Writing – original draft, Writing – review & editing.
